# The Nervous Sequelae of Infectious Diseases1Abstract of a paper read at the meeting of the Medical Society of the County of Erie, held June 21, 1909.

**Published:** 1909-09

**Authors:** James Wright Putnam

**Affiliations:** Buffalo. N. Y.


### The Nervous Sequelae of Infectious Diseases.[1]

BY JAMES WRIGHT PUTNAM, M. D., Buffalo. N. Y.

QUOTING from the statistics of the Massachusetts General Hospital, out-patient department, Dr. Putnam said that in five years there were 5,000 cases of nervous diseases treated. Of these, about 500 had within a year prior to the onset of the illness for which they presented themselves for treatment, some infectious disease which was wholly or partly responsible for the condition. Of these, 80 per cent. were either post-syphilitic or post-influenza. Fifty-three cases were post-diphtheritic, 47 were post-typhoidal, 14 followed scarlet fever, 13 post-malaria, 9 after measles, and 8 after gonorrhea.

Diseases suspected to be of infectious origin, such as beri beri, poliomyelitis, Landry's paralysis, neuritis, myelitis, some of the cerebral palsies of children, chorea, the sclerosis, amputation, neuritis and herpes zoster were among the cases. The vascular lesions as a result of infectious diseases, such as hemorrhages and exudations, and other vasomotor disturbances or changes due to the disturbance of the vessel walls are included.

In variola, where the toxic symptoms of onset were severe, myelitis is the most common sequel. Scarlatina results in optic neuritis and meningitis, measles in optic neuritis and meningitis

1. Abstract of a paper read at the meeting of the Medical Society of the County of Erie, held June 21, 1909.

also. Following typhoid the doctor has seen cases of multiple neuritis, myelitis and poliomyelitis and meningitis. Post-diphtheria, peripheral-neuritis and cord lesions, post-influenza, the sequelæ were multiple neuritis, myelitis, meningitis, encephalitis, neuralgia and retrobulbar neuritis. Following pneumonia they were meningitis and multiple neuritis, after rheumatism, chorea and neuritis. Malaria showed neuritis. Cases of tubercular meningitis and the nervous sequelæ of syphilis, are rather common —the author cited a recent case of motorocular palsy following lues.

Within the past year a case of post-gonorrheal neuritis was seen and successfully treated and in the past month, the author was consulted in the case of multiple neuritis following measles.

